# Exogenous proteinogenic amino acids induce systemic resistance in rice

**DOI:** 10.1186/s12870-016-0748-x

**Published:** 2016-03-03

**Authors:** Naoki Kadotani, Aya Akagi, Hiroshi Takatsuji, Tetsuya Miwa, Daisuke Igarashi

**Affiliations:** Institute for Innovation, Ajinomoto Co., Inc, 1-1, Suzuki-cho, Kawasaki-ku, Kawasaki 210-8681 Japan; Plant Disease Resistance Research Unit, Division of Plant Science, National Institute of Agrobiological Sciences, 2-1-2, Kannondai, Tsukuba, 305-8602 Japan; Bayer Crop Science, Tokyo, 100-8262 Japan

**Keywords:** Amino acid, Glutamate, Disease resistance, Rice, Systemic acquired resistance, Salicylic acid, WRKY45

## Abstract

**Background:**

Plant immune responses can be induced by endogenous and exogenous signaling molecules. Recently, amino acids and their metabolites have been reported to affect the plant immune system. However, how amino acids act in plant defense responses has yet to be clarified. Here, we report that treatment of rice roots with amino acids such as glutamate (Glu) induced systemic disease resistance against rice blast in leaves.

**Results:**

Treatment of roots with Glu activated the transcription of a large variety of defense-related genes both in roots and leaves. In leaves, salicylic acid (SA)-responsive genes, rather than jasmonic acid (JA) or ethylene (ET)-responsive genes, were induced by this treatment. The Glu-induced blast resistance was partially impaired in rice plants deficient in SA signaling such as *NahG* plants expressing an SA hydroxylase, *WRKY45*-knockdown, and *OsNPR1*-knockdown plants. The JA-deficient mutant *cpm2* exhibited full Glu-induced blast resistance.

**Conclusions:**

Our results indicate that the amino acid-induced blast resistance partly depends on the SA pathway but an unknown SA-independent signaling pathway is also involved.

**Electronic supplementary material:**

The online version of this article (doi:10.1186/s12870-016-0748-x) contains supplementary material, which is available to authorized users.

## Background

Twenty proteinogenic amino acids are not only the building blocks of proteins; they or their metabolites also play key roles in development, homeostasis, and growth. In plants, for example, tryptophan is essential for the synthesis of auxins (such as indole-3-acetic acid), which are important growth hormones. Methionine is a precursor of ethylene (ET), an important plant hormone implicated in development and stress signaling. Isoleucine is necessary for the activation of jasmonic acid (JA). Thus, amino acids play essential roles in the regulation of development, growth, and stress responses of plants.

Previous studies revealed the involvement of amino acid metabolism in plant disease responses. In *Arabidopsis thaliana,* inoculation with avirulent *Pseudomonas syringae* pv. *tomato* (*Pto*) expressing *avrRpt2* gene activates the transcription of genes involved in amino acid biosynthesis [1]. Metabolic profiling has also shown that inoculation with virulent or avirulent pathogens alters amino acid contents in *Arabidopsis* [2].

Analysis of *Arabidopsis* mutants strongly supports the hypothesis that amino acids are important for defense responses in plants. The *lht1* (*lys histidine transporter* 1) mutant with reduced levels of Pro, Gln, and Ala shows strong resistance against various types of pathogens such as bacteria, filamentous fungi, and oomycetes [3]. A mutant for the Pro dehydrogenase gene *ProDH* is highly susceptible to avirulent *Pto* AvrRpm1 and has a reduced level of reactive oxygen species (ROS) involved in cell death [4]. These results imply possible involvement of amino acid contents or amino acid metabolism in pathogen susceptibility and defense responses in plants. Despite the apparent relevance of amino acid metabolism to disease resistance, the details of these phenomena have yet to be elucidated.

Multiple signaling pathways mediated by plant hormones, such as salicylic acid (SA), JA, and ET, play a role in disease resistance. The SA pathway has been extensively investigated over the last decades, especially with respect to its role in systemic acquired resistance (SAR) in dicots. In Arabidopsis, *Nonexpressor of PR1* (*NPR1*) plays key roles in SAR [5]. In rice, the SA pathway branches into two sub-pathways, which are dependent on (co-) transcription factors *OsNPR1* or *OsWRKY45*, respectively [6–9]. *OsWRKY45* is essential for SA- and benzothiadiazole (BTH)-induced resistance against rice blast and bacterial blight diseases [10, 11]. BTH-induced blast resistance is impaired in both *OsNPR1*-knockdown (−kd) and *OsWRKY45*-kd rice lines [10, 11].

JA and ET are also involved in rice resistance against blast disease. For example, systemic resistance against rice blast induced by root treatment with *Pseudomonas fluorescens* WCS374r is SA-independent, and JA- and ET-dependent [12]. Rice *COLEOPTILE PHOTOMORPHOGENESIS 2* (*CPM2)* is a single-copy gene for allene oxide cyclase (AOC), which is essential for JA synthesis. Riemann et al. reported that a *cpm2* mutant, the JA concentration in which is extremely low, is sensitive to an otherwise incompatible strain of *Magnaporthe oryzae* [13]. Overexpression of *OsAOS2* encoding an allene oxide synthase elevated the JA concentration and enhanced rice resistance against *M. oryzae* [14]. Overexpression of *OsACS2* encoding a 1-aminocyclopropane-1-carboxylate synthase elevated the ET concentration and consequently enhanced resistance against *M. oryzae* in rice [15]. These investigations imply that multiple signaling pathways may be activated in response to rice blast in rice.

Previously, we reported that glutamate fermentation byproducts (GFB) confer resistance to pathogen infection in *Arabidopsis* [16]. In this study, we aimed to clarify the role of amino acids in rice defense responses and found that treating roots with amino acids induces systemic defense responses and suppresses infection of compatible rice with blast fungus. We showed that the SA signaling pathway and at least one other unknown pathway are involved in this systemic resistance. These findings provide a new perspective on understanding the mechanisms of plant immunity and rice protection from infectious diseases.

## Results

**Fig. 1:**
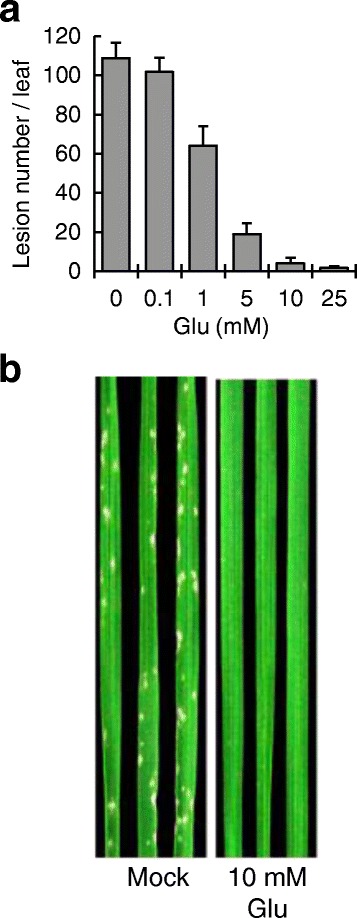
Glu concentration–dependent reduction of blast lesions. **a**, Rice plants were grown in growth chambers, dipped into Glu solution from the roots for 24 h, and inoculated with *Magnaporthe oryzae*. Lesions on the fourth leaves were counted 7 d after inoculation. The data are means ± SE (*n* = 6). **b**, Blast lesions on the fourth leaves

To investigate the effect of Glu on disease resistance, we immersed rice roots into 0.1–25 mM Glu solutions for 24 h, sprayed *M. oryzae* conidia onto rice plants, and analyzed disease symptoms 5 d after treatment. Glu treatment markedly reduced blast lesions on leaves in a concentration-dependent manner (Fig. 1). This result indicates an induction of systemic disease resistance. Because Glu concentrations above 10 mM suppressed plant growth, we used 10 mM Glu in the following experiments.

**Fig. 2:**
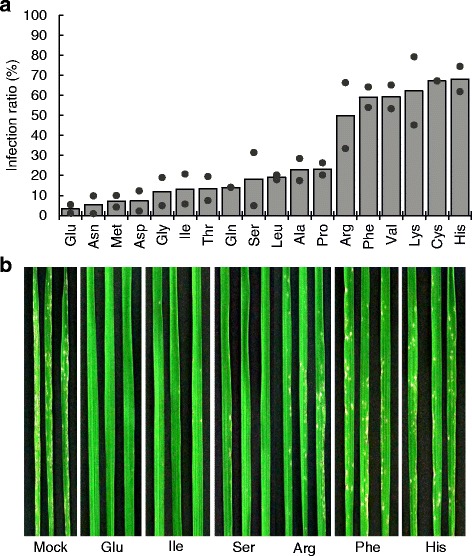
Rice blast resistance induced by amino acids. **a**, The infection ratio in amino acid-treated rice. Roots were dipped into 10 mM L-amino acid solutions for 24 h and inoculated with rice blast fungus. Blast lesions were counted 5 d later and the ratios between the values for mock-inoculated and fungus-inoculated plants were calculated. The experiment was repeated twice. Circles indicate the infection ratio obtained from two independent experiments and the bars show the average values. **b**, Blast lesions on rice leaves treated with amino acids

To test the effects of other amino acids, we performed a similar experiment with 18 amino acids (10 mM; Trp and Tyr were excluded because of their low solubility in water). We found that all amino acids conferred resistance (Fig. 2). In addition to Glu, three other amino acids, Asn, Met, and Asp, induced strong resistance. In contrast, basic amino acids (Arg, Lys, and His), some hydrophobic amino acids (Phe and Val) and Cys induced weak resistance. Hydrophobicity does not appear to be a determinant because other hydrophobic amino acids, such as Ile and Leu, induced substantial resistance. These results suggest that amino acids have potential to trigger resistance against rice blast. We observed some growth inhibition when roots were treated with Met or Ile, but not with other amino acids (data not shown).

**Fig 3:**
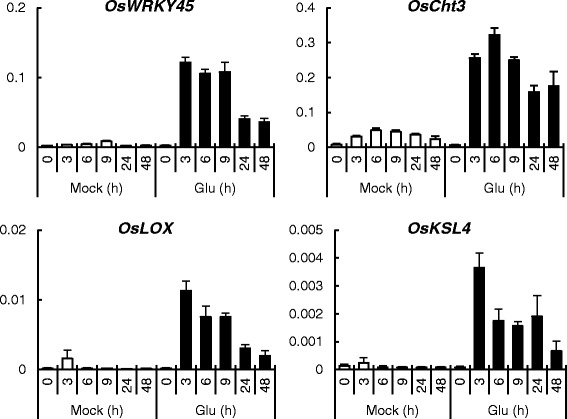
Temporal expression patterns of defense-related genes in the roots of Glu-treated rice. Roots were treated with water (mock) or 10 mM Glu for the indicated periods of time. Total RNA was extracted from roots, and the expression of the genes of interest was analyzed by qRT-PCR. Y-axis shows relative mRNA expression to *eEF-1a*. The data are means ± SE (*n* = 3)

To assess Glu-induced defense responses, we performed microarray analysis of the fourth leaves of 2-week-old plants the roots of which were treated with Glu for 8 or 24 h (Additional file 1). This analysis indicated that Glu treatment increased the transcript levels of Several SA-responsive genes, such as *OsWRKY76, OsWRKY19, OsWRKY45*, *SA-glucosyltransferase*, *GST*, and *OsPR1b* (Table 1) and several JA-responsive genes, such as *OsJAZ3* and *OsJAZ4* were upregulated (Table 2). To investigate the early events induced by Glu, we analyzed the expression patterns of defense-related genes by qRT-PCR in roots (Fig. 3). Several genes rapidly responded to Glu, and their transcript levels reached maximum within 6 h after treatment.

**Table 1:** Changes in the expression of SA-dependent genes upon Glu treatment detected by microarray analysis

Gene	GenBank accession	Locus ID	Fold change (log ratio)	
			8 h	24 h
*Cytochrome P450*	AK072220	Os07g0418500	−0.02	−1.35
*GST (Tau class)*	AK103453	Os10g0528300	−1.3	61.28
*OsLOX*	AK072241	Os12g0559200	−1.21	1.72
*OsPR-1a*	AF251277	Os07g0129200	−1.27	−1.21
*OsPR-1b*	AK107926	Os01g0382000	4.55	18.16
*OsWRKY19*	AK108389	Os05g0571200	13.21	6.26
*OsWRKY45*	AK066255	Os05g0322900	8.08	7.74
*OsWRKY62*	AK067834	Os09g0417800	−1.02	−1.35
*OsWRKY76*	AK068337	Os09g0417600	7.19	1.78
*PBZ1*	AK071613	Os12g0555500	0.001	−2.05
*SA-glucosyltransferase*	AK064395	Os09g0518200	−0.009	22.18

**Table 2:** Changes in the expression of JA-dependent genes upon Glu treatment detected by microarray analysis

Gene	GenBank accession	Locus ID	Fold change (log ratio)	
			8 h	24 h
*OsJAZ2*	AK073589	Os03g0180900	1.13	−1.46
*OsJAZ3*	AK070649	Os03g0180800	2.14	−1.88
*OsJAZ4*	AK120087	Os03g0181100	5.84	−1.26
*OsJAZ5*	AK061842	Os03g0402800	1.95	1.6
*OsJAZ11*	AK107750	Os04g0395800	1.67	−1.35
*OsbHLH148*	AK071734	Os03g0741100	1.44	1.08

**Fig 4:**
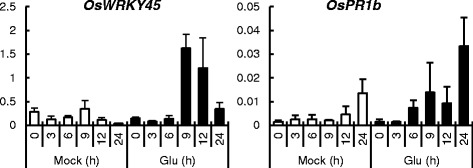
Temporal expression patterns of defense-related genes in the fourth leaves of Glu-treated rice. Roots were treated with water (mock) or 10 mM Glu for the indicated periods of time. Total RNA was extracted from the fourth leaves, and the expression of the genes of interest was analyzed by qRT-PCR. Y-axis shows relative mRNA expression to *UBQ*. The data are means ± SE (*n* = 3)

In our qRT-PCR analysis of several pathogen-responsive genes in the fourth leaves, the expression of the SA-responsive genes *OsWRKY45* and OsPR1b were remarkably up-regulated by Glu treatment (Fig. 4). These results suggest that Glu treatment induces rapid responses in roots, mainly in SA signaling, and the signal is then transmitted to leaves, where it activates SA-responses.

**Fig 5:**
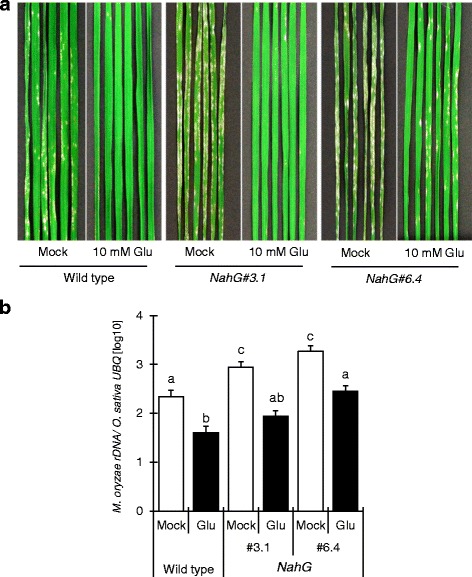
Glu-induced rice blast resistance in lines #3.1 and #6.4 of *NahG* transgenic rice. **a**, Blast lesions on the fourth leaves of wild-type and *NahG* rice treated with water (mock) or 10 mM Glu. **b**, The ratio of the level of *M. oryzae* 28S rDNA to that of rice *UBQ* in infected leaves. The data are means ± SE (*n* = 12); Different letters indicate significant differences as determined by ANOVA followed by Tukey’s multiple comparison test (*P* < 0.05)

**Fig 6:**
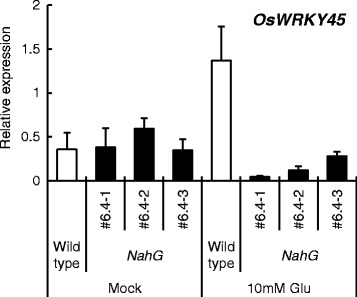
*OsWRKY45* expression in wild-type rice and three lines of *NahG* rice. Roots were treated with water (mock) or 10 mM Glu for 24 h, and *OsWRKY45* expression in the fourth leaves was analyzed by qRT-PCR. Y-axis shows relative mRNA expression to *UBQ.* The data are means ± SE (*n* = 5)

To investigate the contribution of SA signaling to Glu-induced rice blast resistance, we used two lines (#3.1 and #6.4) of SA-deficient *NahG* transgenic plants; *NahG* plants are more susceptible to rice blast than wild-type because of the decrease in SA [17]. The plants were treated with Glu for 24 h and inoculated with *M. oryzae*. Glu suppressed blast symptoms drastically in both *NahG* lines, although #6.4 was more susceptible than #3.1 (Fig. 5a), indicating that Glu-induced blast resistance occurs even in SA-deficient plants. The levels of *M. oryzae* 28S rDNA were consistent with the severity of blast symptoms (Fig. 5b). Using *NahG* plants (#6.4), we also determined the effect of Glu on the expression of *OsWRKY45* (Fig. 6). A Glu-induced increase in *OsWRKY45* expression was observed in wild-type leaves but was barely noticeable in *NahG* leaves (Fig. 6). These results suggest that the SA pathway is involved in Glu-induced resistance but its contribution is partial.

**Fig 7:**
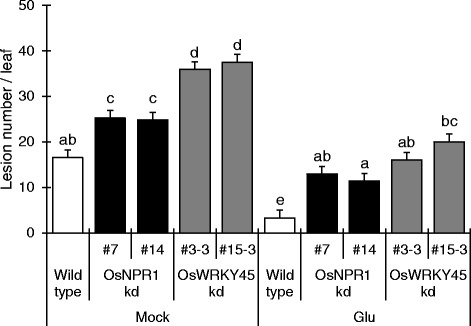
Effects of Glu on blast resistance of OsWRKY45-kd and OsNPR1-kd rice. The number of lesions on the fourth leaves of the rice treated with water (mock) or 10 mM Glu was counted 5 d after inoculation. The data are means ± SE (*n* = 10); Different letters indicate significant differences as determined by ANOVA followed by Tukey’s multiple comparison test (*P* < 0.05)

It has previously been shown that BTH-induced blast resistance is compromised in *OsNPR1*-kd [7] and *OsWRKY45*-kd [10] rice plants. To further investigate the involvement of the SA pathway, we used *OsNPR1*-kd lines (#7 and #14) and *OsWRKY45*-kd lines (#3-3 and #15-3) (Fig. 7). Mock-treated *OsNPR1*-kd and *OsWRKY45*-kd plants were more susceptible to rice blast than were wild type, indicating that basal defense is impaired in these lines. Glu treatment strongly induced blast resistance in wild type. In *OsNPR1*-kd and *OsWRKY45*-kd plants, Glu treatment induced blast resistance in comparison with mock treatment. However, Glu-treated *OsNPR1*-kd and *OsWRKY45*-kd plants showed disease symptoms comparable to those in mock-treated wild type. Taken together, these results indicate that Glu-induced blast resistance is partially dependent on SA signaling, but an unknown SA-independent pathway is also involved.

**Fig 8:**
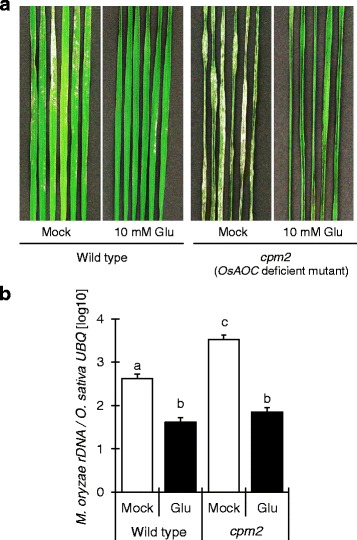
The effect of JA on Glu-induced rice blast resistance in wild-type and a *cpm2* mutant rice. **a**, Blast lesions on the fourth leaves of wild-type rice and a *cpm2* mutant treated with water (mock) or 10 mM Glu. **b**, The ratio of the level of *M. oryzae* 28S rDNA to that of rice *UBQ* in infected leaves The data are means ± SE (*n =* 12). Different letters indicate significant differences as determined by ANOVA followed by Tukey’s multiple comparison test (*P* < 0.05)

Since JA also plays a role in rice blast resistance, we also examined whether it is involved in Glu-induced resistance by examining the JA-deficient *cpm2* mutant. Glu induced blast resistance both in *cpm2* and wild type (Fig. 8), indicating that Glu responses are independent of JA.

**Fig 9:**
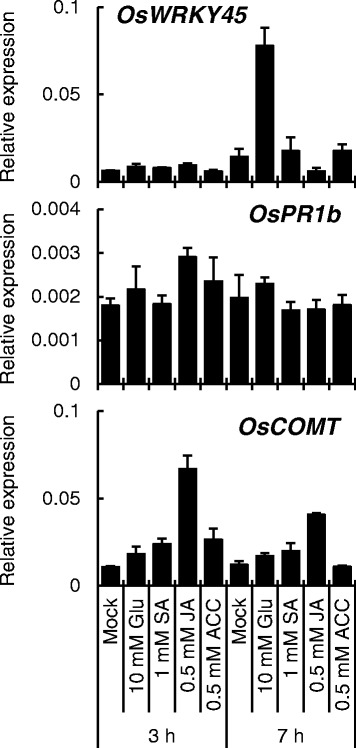
The effect of plant hormones on induced systemic resistance. Roots were treated with water (mock) or each compound, and the expression of the genes in the fourth leaves was analyzed by qRT-PCR. Y-axis shows relative mRNA expression to *UBQ.* The data are means ± SE (*n* = 3)

To examine whether ET is involved in Glu-induced disease resistance, we performed gene expression analysis of *OsWRKY45*, *OsPR1b* and *OsCOMT* 3 or 7 h after root treatment with SA, JA, ACC, or Glu. The transcript level of *OsWRKY45* was markedly elevated 7 h after Glu treatment, whereas no increase was observed upon ACC treatment (Fig. 9), consistent with a previous report [10]. These results suggest that the ET pathway is not important for Glu-induced resistance. JA-responsive *OsCOMT1* was not induced by Glu treatment, consistent with the result that Glu-induced blast resistance was not impaired in *cpm2*. Interestingly, 1 mM SA did not induce *OsWRKY45 and OsPR1b* expression in the fourth leaves, suggesting that SA synthesis in the root is not important for Glu-induced resistance.

## Discussion

There is a report indicating that the amino acid metabolism in plant cells changes when the plant is infected with pathogens [2]. In Arabidopsis mutants with altered amino acid metabolism, such as *lht1*, primary metabolites, including amino acids, affect the plant–microbe interaction, and an association between amino acid metabolism and the SA pathway has been suggested [3]. The *LHT1* gene was upregulated by pathogen infection in wild type but not in a *NahG* transformant and SA pathway–deficient mutants such as *pad4*, *sid2* and *npr1* [3]. The increased expression of the *Pto* AvrRpm1–induced *ProDH* gene encoding a Glu-generating enzyme depends on the exogenous SA concentration and is markedly lower in *sid2* and *npr1* than in wild type [4]. In rice, the effect of Glu was decreased in *OsWRKY45*-kd, *OsNPR1*-kd, and *NahG* transformants; therefore, the SA pathway is most likely involved in Glu-induced resistance. However, the decrease of the induction of resistance by Glu in each transformant was incomplete. Therefore, it is also likely that a molecule (s) other than SA mediates systemic resistance induced by Glu treatment.

Glu might be recognized by a glutamate receptor–like (GLR) protein (s). Arabidopsis has 20 genes encoding GLRs that share considerable similarity in primary and predicted secondary structures with animal ionotropic glutamate receptor (iGluR) subunits [18]. Some plant iGluR homologs have been implicated in root development, ion transport, and several metabolic and signaling pathways [19]. Arabidopsis expressing radish *GLR* cDNA shows an increased expression of defense-related genes and enhanced resistance to *Botrytis cinerea* [20]. The *AtGLR3.3* mutant is more susceptible to *Hyaloperonospora arabidopsidis* and *Pseudomonas syringae* than wild type and fails to activate the transcription of defense-related genes [21]. Recent studies have shown that GLR have broad specificities to amino acids [22]. This is consistent with our observation that various amino acids induced blast resistance at different extents. Hence, initial cellular event in the amino–acid–induced systemic resistance could involve GLRs.

The simplest model of defense signal transduction from roots to leaves is that Glu or its metabolite is transported to the leaves directly. However, Glu treatment of roots did not significantly change the content of free amino acids in leaves (Additional file 2). If GLRs are involved in the recognition of exogenous amino acids, electrical signal should be transmitted from roots to leaves. In Arabidopsis, several GLRs functions in agonist–stimulated plasma membrane depolarization and can control cytosolic Ca^2+^ influxes [23]. AtGLR 3.3 has also been implicated in the defense response to mechanical wounding and systemic wound–induced gene expression, and electrical signal transmission is eliminated in *atglr 3.3* and *atglr 3.6* double mutant [24]. Various novel signal molecules have been reported [25]. One of these is pipecolic acid (Pip), an amino acid synthesized from Lys and required for systemic acquired resistance (SAR) in Arabidopsis [26]. There are several similarities between Pip- and Glu-induced disease resistance at the point that amino acid induced disease resistance mediated by SA-pathway. Our results, however, demonstrate that Lys, the direct precursor of Pip, induced much weaker resistance than Glu. Another defense-inducing amino acid is Pro. Free Pro accumulation has been observed in response to environmental stresses, including pathogen attack [27]. Pro is converted to Glu by two reactions catalyzed by two enzymes, ProDH and Δ^1^-pyrroline-5-carboxylate dehydrogenase (P5CDH). ROS generated in this catabolic pathway contribute to hypersensitive response and disease resistance [4]. In this study, however, we found that Pro did not induce strong resistance compared to Glu. If ProDH induces systemic resistance in rice, the effect of Glu should be weaker than that of Pro. Therefore, the Glu-induced systemic resistance in rice is unlikely to be mediated by ROS derived from Pro catabolism.

The defense response requires amino acids as a resource for synthesis of defense proteins. Therefore, Glu and other amino acids may induce SA-independent resistance by contributing to a pool of amino acids. Glu is connected to the TCA cycle by glutamate dehydrogenase and therefore could serve as a source of energy and carbon for synthesis of defense compounds such as phytoalexins. This pathway could contribute to SA-independent defense.

## Conclusions

In this study, we demonstrated that exogenous application of free amino acids induces blast resistance in rice. Systemic induction of the SA-dependent defense response is most likely involved, but another unknown mechanism also appears to be involved in this phenomenon. Although we could not completely elucidate the mechanism of disease resistance induced by Glu and other amino acids, our findings partly explain the induction of disease resistance by GFB in several crops [16]. This knowledge may contribute to the development of agricultural materials or farming systems with reduced environmental loads.

## Methods

### Plant materials, growth conditions, and inoculation assay

All experiments were performed in rice (*Oryza sativa* cv. Nipponbare or cv. Nihonmasari) at the 4-leaf stage. OsWRKY45-kd [10], OsNPR1-kd [7] and *NahG* [17] transgenic plants were generated from Nipponbare as described. Nihonmasari was used only for the analysis of *cpm2* mutant analysis. The *cpm2* mutant was isolated from γ–ray-mutagenized M2 lines of Nihonmasari as described by Biswas et al. [28]. Unless otherwise noted, plants were cultivated in growth chambers under a 16 h light, 28 °C / 8 h dark, 25 °C regime, light intensity of 200 μmol m^−2^s^−1^, and 70 % humidity. Roots were treated by dipping with 10 mM L-amino acid, 1 mM SA, 0.5 mM JA, or 0.5 mM ACC (50 mL/plant). In *M. oryzae* inoculation assays, one of the eighteen amino acids (Glu-Na, Ala, Asp-Na, Cys-HCl, Phe, Gly, His, Ile, Lys-HCl, Leu, Met, Asn, Pro, Gln, Arg, Ser, Thr, or Val; commercially available products) was used. Water was used in mock treatments.

Conidia of rice blast fungus, Japanese isolate KEN53-33 of *M. oryzae*, were suspended in 0.01 % Tween 20 at a density of 10^5^/mL and sprayed onto rice plants at the four-leaf stage as described [29, 30]). Disease symptoms were evaluated by counting lesions or determining fungal *28S* rDNA content in the fourth leaves by qPCR as described [31]. Fungal growth was evaluated from the ratio of *M. oryzae* 28S rDNA to rice *ubiquitin 1* (*UBQ*) DNA.

### Real-time PCR and microarray analysis

Roots were dipped in a solution containing an amino acid or plant hormone. Roots and fourth leaves harvested from 2 plants were pooled, and total RNA was isolated from each pool using an RNeasy plant mini kit (Qiagen). cDNA was generated from 0.5 μg of total RNA with a ReverTra Ace qPCR RT Master Mix reagent kit (Toyobo) according to the manufacturer’s instructions. qPCR was performed on an ABI 7500 Fast Real-time PCR System with ABI Fast SYBR Green Master Mix (Life Technologies). Amplification was performed by two-step PCR with 40 cycles of denaturation at 95 °C for 3 s and extension/detection at 60 °C for 30 s. *UBQ* (Shimono et al. 2007) and *elongation factor 1a* (*eEF-1a*) [32] were used as internal standards for leaves and roots, respectively. Primer sets for PCR (Additional file 3) were tested by dissociation curve analysis and verified to confirm that the absence of non-specific amplification. Gene names which we analyzed by qPCR, are listed in Additional file 3.

For microarray analysis, roots were treated with Glu solution or water as mock, fourth leaves were harvested and pooled as described above. Total RNA was isolated by using an RNeasy plant mini kit (Qiagen). Microarray analysis was performed as described [10] (Shimono et al. 2007).

### Availability of supporting data

The data sets supporting the results of this article are included within the article and its additional files. The microarray data supporting the results of this article are available in the NCBI’s Gene Expression Omnibus repository, and are accessible through GEO Series accession number GSE78266 (http://www.ncbi.nlm.nih.gov/geo/query/acc.cgi?acc=GSE78266).

## Additional files

Additional file 1: Raw microarray data from the leaf by root treatment with Glu. (XLSX 4942 kb) Additional file 2: Concentrations of amino acids in rice leaves. (PDF 187 kb) Additional file 3: List of PCR primers. (PDF 79 kb)

## Abbreviations

CPM: coleoptile photomorphogenesis
ET: ethylene
JA: jasmonic acid
NahG: NPR: nonexpressor of PR1
PR: pathogenesis related
SA: salicylic acid

## References

[1] Scheideler M, Schlaich NL, Fellenberg K, Beissbarth T, Hauser NC, Vingron M (2002). Monitoring the switch from housekeeping to pathogen defense metabolism in *Arabidopsis* thaliana using cDNA arrays. J Biol Chem.

[2] Ward JL, Forcat S, Beckmann M, Bennett M, Miller SJ, Baker JM (2010). The metabolic transition during disease following infection of Arabidopsis thaliana by *Pseudomonas syringae* pv *tomato*. Plant J.

[3] Liu G, Ji Y, Bhuiyan NH, Pilot G, Selvaraj G, Zou J (2010). Amino acid homeostasis modulates salicylic acid-associated redox status and defense responses in Arabidopsis. Plant Cell.

[4] Cecchini NM, Monteoliva MI, Alvarez ME (2011). Proline dehydrogenase contributes to pathogen defense in Arabidopsis. Plant Physiol.

[5] Cao H, Glazebrook J, Clarke JD, Volko S, Dong X (1997). The Arabidopsis NPR1 gene that controls systemic acquired resistance encodes a novel protein containing ankyrin repeats. CELL.

[6] Nakayama A, Fukushima S, Goto S, Matsushita A, Shimono M, Sugano S (2013). Genome-wide identification of WRKY45-regulated genes that mediate benzothiadiazole-induced defense responses in rice. BMC Plant Biol.

[7] Sugano S, Jiang CJ, Miyazawa S, Masumoto C, Yazawa K, Hayashi N, Shimono M, Nakayama A, Miyao M, Takatsuji H (2010). Role of OsNPR1 in rice defense program as revealed by genome-wide expression analysis. Plant Mol Biol.

[8] Takatsuji H, Jiang CJ, Sugano S (2010). Salicylic acid signaling pathway in rice and the potential applications of its regulators. JARQ.

[9] Takatsuji H (2014). Development of disease-resistant rice using regulatory components of induced disease resistance. Front Plant Sci.

[10] Shimono M, Sugano S, Nakayama A, Jiang CJ, Ono K, Toki S (2007). Rice WRKY45 plays a crucial role in benzothiadiazole-inducible blast resistance. Plant Cell.

[11] Shimono M, Koga H, Akagi A, Hayashi N, Goto S, Sawada M (2012). Rice WRKY45 plays important roles in fungal and bacterial disease resistance. Mol Plant Pathol.

[12] De Vleesschauwer D, Djavaheri M, Bakker PA, Höfte M (2008). *Pseudomonas fluorescens* WCS374r-induced systemic resistance in rice against *Magnaporthe oryzae* is based on pseudobactin-mediated priming for a salicylic acid-repressible multifaceted defense response. Plant Physiol.

[13] Riemann M, Haga K, Shimizu T, Okada K, Ando S, Mochizuki S (2013). Identification of rice Allene Oxide Cyclase mutants and the function of jasmonate for defence against *Magnaporthe oryzae*. Plant J.

[14] Mei C, Qi M, Sheng G, Yang Y (2006). Inducible overexpression of a rice allene oxide synthase gene increases the endogenous jasmonic acid level, PR gene expression, and host resistance to fungal infection. Mol Plant-Microbe Interact.

[15] Helliwell EE, Wang Q, Yang Y (2013). Transgenic rice with inducible ethylene production exhibits broad-spectrum disease resistance to the fungal pathogens *Magnaporthe oryzae* and Rhizoctonia solani. Plant Biotechnol J.

[16] Igarashi D, Takeda T, Narusaka Y, Totsuka K (2010). Glutamate fermentation by-product activates plant defense responses and confers resistance against pathogen infection. J Phytopathol.

[17] Yang Y, Qi M, Mei C (2004). Endogenous salicylic acid protects rice plants from oxidative damage caused by aging as well as biotic and abiotic stress. Plant J.

[18] Lam HM, Chiu J, Hsieh MH, Meisel L, Oliveira IC, Shin M (1998). Glutamate-receptor genes in plants. Nature.

[19] Tapken D, Anschütz U, Liu LH, Huelsken T, Seebohm G, Becker D (2013). A plant homolog of animal glutamate receptors is an ion channel gated by multiple hydrophobic amino acids. Sci Signal.

[20] Kang S, Kim HB, Lee H, Choi JY, Heu S, Oh CJ (2006). Overexpression in Arabidopsis of a plasma membrane-targeting glutamate receptor from small radish increases glutamate-mediated Ca^2+^ influx and delays fungal infection. Mol Cells.

[21] Manzoor H, Kelloniemi J, Chiltz A, Wendehenne D, Pugin A, Poinssot B (2013). Involvement of the glutamate receptor AtGLR33 in plant defense signaling and resistance to *Hyaloperonospora arabidopsidis*. Plant J.

[22] Forde BG, Roberts MR (2014). Glutamate receptor-like channels in plants: a role as amino acid sensors in plant defence?. F1000Prime Rep.

[23] Stephens NR, Qi Z, Spalding EP (2008). Glutamate receptor subtypes evidenced by differences in desensitization and dependence on the GLR33 and GLR34 genes. Plant Physiol.

[24] Mousavi SA, Chauvin A, Pascaud F, Kellenberger S, Farmer EE (2013). GLUTAMATE RECEPTOR-LIKE genes mediate leaf-to-leaf wound signaling. Nature.

[25] Dempsey DA, Klessig DF (2012). SOS – too many signals for systemic acquired resistance?. Trends Plant Sci.

[26] Návarová H, Bernsdorff F, Döring AC, Zeier J (2012). Pipecolic acid, an endogenous mediator of defense amplification and priming, is a critical regulator of inducible plant immunity. Plant Cell.

[27] Fabro G, Kovács I, Pavet V, Szabados L, Alvarez ME (2004). Proline accumulation and AtP5CS2 gene activation are induced by plant-pathogen incompatible interactions in Arabidopsis. Mol Plant-Microbe Interact.

[28] Biswas KK, Neumann R, Haga K, Yatoh O, Iino M (2003). Photomorphogenesis of rice seedlings: a mutant impaired in phytochrome-mediated inhibition of coleoptile growth. Plant Cell Physiol.

[29] Hirata K, Kusaba M, Chuma I, Osue J, Nakayashiki H, Mayama S (2007). Speciation in Pyricularia inferred from multilocus phylogenetic analysis. Mycol Res.

[30] Watanabe T, Igarashi H, Matsumoto K, Seki S, Mase S, Sekizawa Y (1977). The characteristics of probenazole (Oryzemates®) for the control of rice blast. J Pestic Sci.

[31] Qi M, Yang Y (2002). Quantification of *Magnaporthe grisea* during infection of rice plants using real-time polymerase chain reaction and northern blot/phosphoimaging analyses. Phytopathology.

[32] Jain M, Nijhawan A, Tyagi AK, Khurana JP (2006). Validation of housekeeping genes as internal control for studying gene expression in rice by quantitative real-time PCR. Biochem Biophys Res Commun.

