# Supplementary material for: Exogenous proteinogenic amino acids induce systemic resistance in rice
Source: BMC Plant Biol. 2016 Mar 3;16:60. doi: 10.1186/s12870-016-0748-x (PMC4778346; doi:10.1186/s12870-016-0748-x)
Supplement: Additional file 2: — Concentrations of amino acids in rice leaves. (PDF 187 kb) [file 12870_2016_748_MOESM2_ESM.pdf]

Additional file 2.

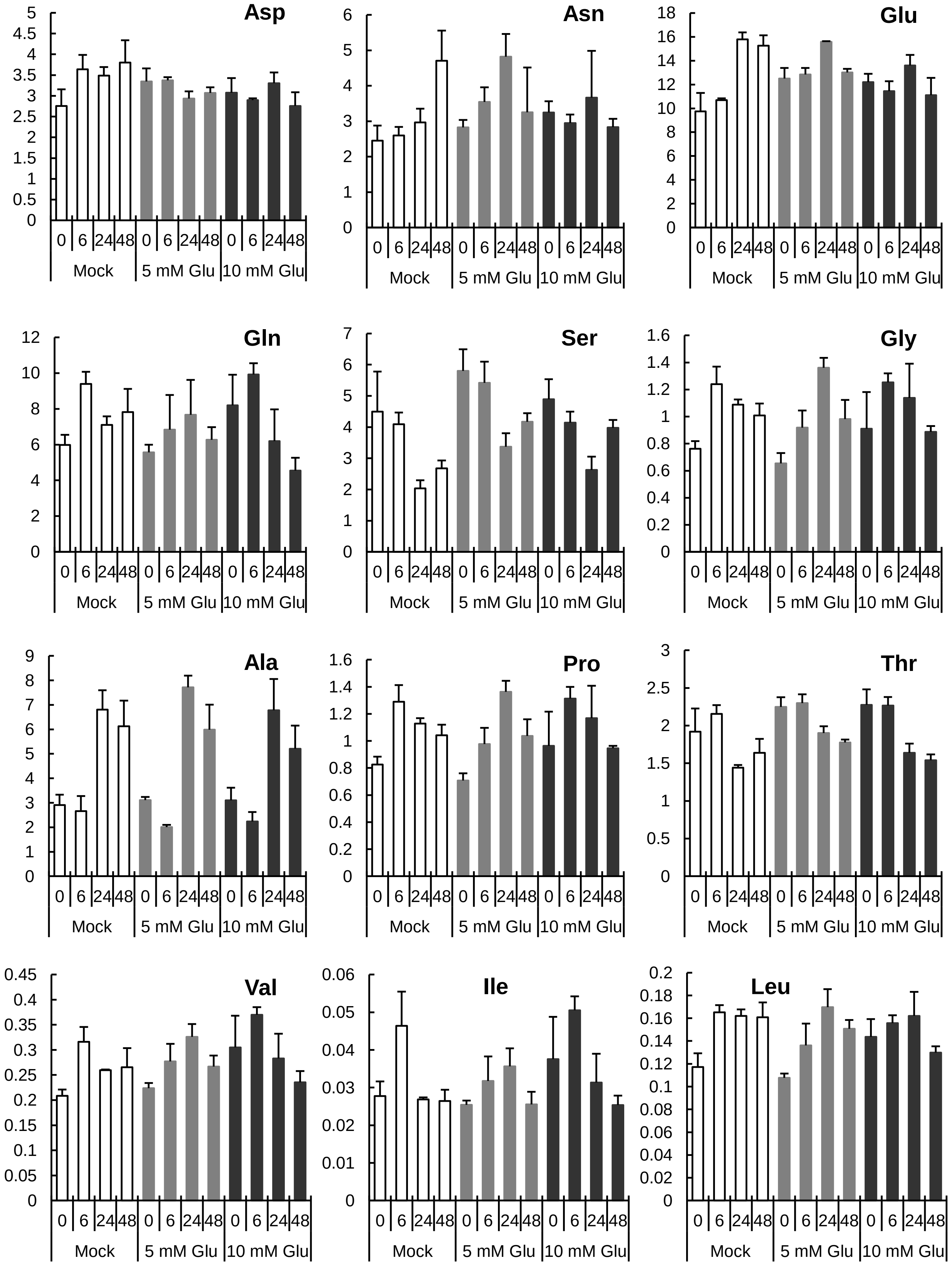

Additional file 2.

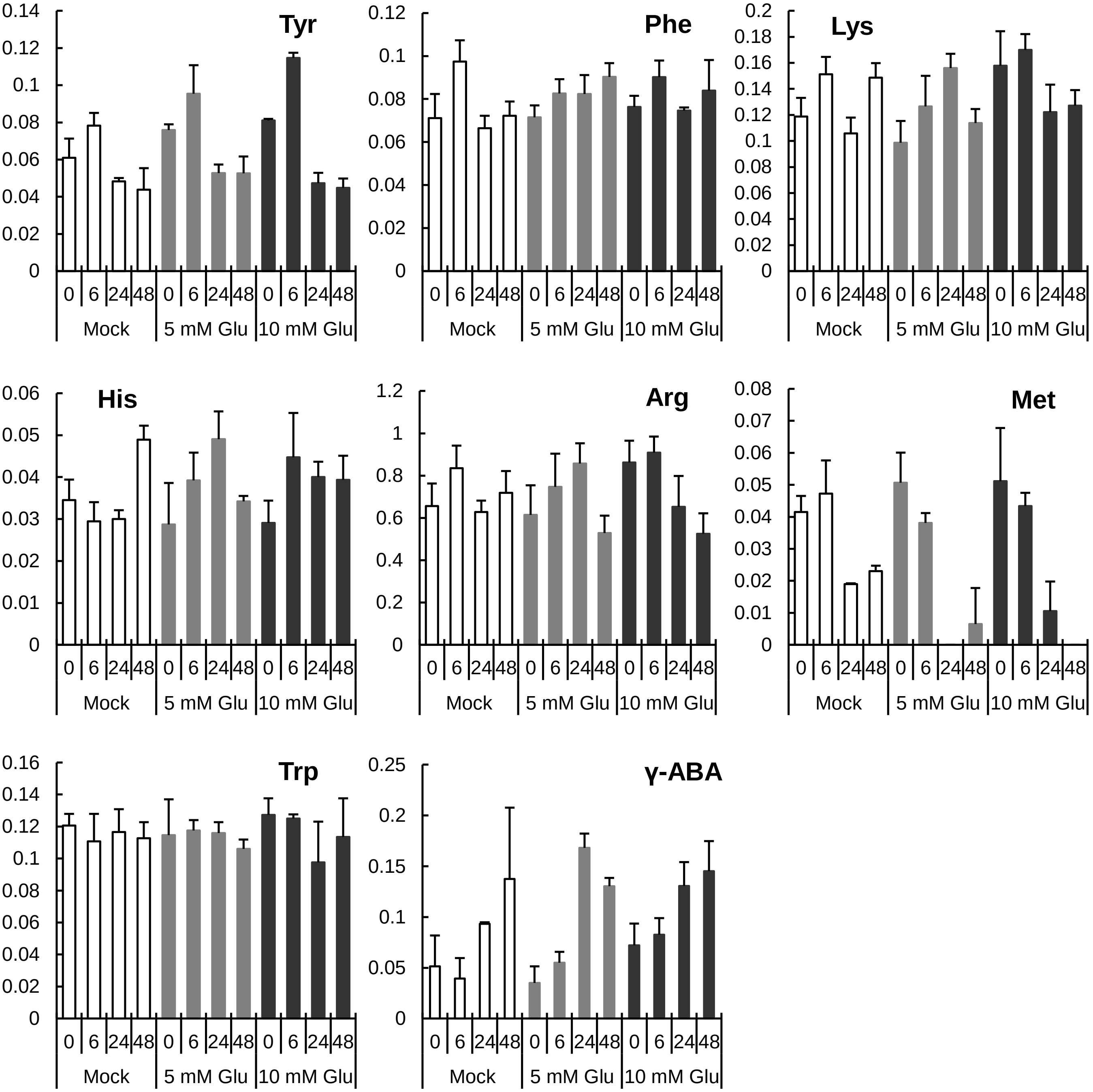

Additional file 2. Concentrations of amino acids in rice leaves. Because immersion of rice roots in Glu solution triggered systemic disease resistance, we thought that some signal may be transmitted to the leaves from the roots. The simplest model is that Glu absorbed by roots is circulated into the leaves. To test this possibility, we quantified the concentrations of 20 amino acids and γ-ABA by using an amino acid analyzer in fourth leaves of plants treated with water (mock), 5 mM Glu, or 10 mM Glu for 0, 6, 24, or 48 h. Y-axis shows contents of each amino acid (nmol/mg fresh weight). The data are means ±SD (n=3). No significant differences were observed between mock and Glu treatment. These results indicate that the molecule that induces systemic resistance in rice is not Glu or a proteinogenic amino acid derived from Glu.
Amino acid content was determined using an L-8800 amino acid analyzer (Hitachi) as described (Igarashi et al. 2006).

Du, B., Zhang, W., Liu, B., Hu, J., Wei, Z., Shi, Z., He, R., Zhu, L., Chen, R., Han, B., and He, G. 2009. Identification and characterization of Bph14, a gene conferring resistance to brown planthopper in rice. Proc. Natl. Acad. Sci. U. S. A. 106:22163-22168.

Igarashi, D., Tsuchida, H., Miyao, M., and Ohsumi, C. 2006. Glutamate:glyoxylate aminotransferase modulates amino acid content during photorespiration. Plant Physiol. 142:901-910.

Okada, A., Okada, K., Miyamoto, K., Koga, J., Shibuya, N., Nojiri, H., and Yamane, H. 2009. OsTGAP1, a bZIP transcription factor, coordinately regulates the inductive production of diterpenoid phytoalexins in rice. J. Biol. Chem. 284:26510-26518.
