# Supplementary material for: Exogenous proteinogenic amino acids induce systemic resistance in rice
Source: BMC Plant Biol. 2016 Mar 3;16:60. doi: 10.1186/s12870-016-0748-x (PMC4778346; doi:10.1186/s12870-016-0748-x)
Supplement: Additional file 3: — List of PCR primers. (PDF 79 kb) [file 12870_2016_748_MOESM3_ESM.pdf]

Additional file 3. PCR primers

Gene	Gene name / function	Locus ID	Forward primer (5′-3′)	Reverse primer (5′-3′)	Reference
OsWRKY45	*Transcription factor*	Os05g0322900	CGGGTAAAACGATCGAAAGA	TTTCGAAAGCGGAAGAACAG	Shimono et al. 2007
OsPR1b	*Pathogenesis related protein*	Os01g0382000	ACGGGCGTACGTACTGGCTA	CTCGGTATGGACCGTGAAG	Shimono et al. 2007
UBQ	*Ubiquitin*	Os06g0681400	GTGGTGGCCAGTAAGTCCTC	GGACACAATGATTAGGGATCA	Shimono et al. 2007
OsCOMT1	*Caffeic acid O-methyltransferase*	Os08g0157500	AGGTGTTCGACCATCGTCTT	CACCGGAATTGAACATCAAA	Shimizu et al.. 2012
eEF-1a	*Elongation factor*	Os03g0177500	TTTCACTCTTGGTGTGAAGCAGAT	GACTTCCTTCACGATTTCATCGTAA	Jain et al. 2006
OsKSL4	*kaurene synthase-like*	Os04g0179700	CGCCTTTGTAACTCTAAGGTA	ACGTAAAAGGCTTGTATATC	Okada et al. 2009
OsCht3	*Chitinase*	Os06g0726100	AGCTATGGCGATAACCTGGA	TCGTCTGCTCGGATCAAATA	This study
